# Supplementary material for: On the performance of a photosystem II reaction centre-based photocell
Source: Chem Sci. 2017 Aug 4;8(10):6871–80. doi: 10.1039/c7sc02983g (PMC5636947; doi:10.1039/c7sc02983g)
Supplement: Supplementary file 1 [file SC-008-C7SC02983G-s001.pdf]

Electronic Supplementary Material (ESI) for Chemical Science.
This journal is © The Royal Society of Chemistry 2017

Supplementary information for "On the performance of a photosystem II reaction centre-based photocell"

Richard Stones, Hoda Hossein-Nejad, Rienk van Grondelle, Alexandra Olaya-Castro

July 30, 2017

Supplementary Note 1: PSIIRC parameters

Electronic Parameters

The state energies in wavenumbers for the photocell model are given in Supplementary Table S1. The electronic PSIIRC Hamiltonian H_{el} used for the excited state dynamics can be constructed using the energies of the single excitation states of the six core chromophores and two charge transfer (CT) states of the Chl_{D1} charge separation pathway[26] along with the couplings from Supplementary Table S2.

g	P_{D1}	P_{D2}	Chl_{D1}	Chl_{D2}	Phe_{D1}	Phe_{D2}	$\mathrm{Chl}^{+}_{D1}\mathrm{Phe}^{-}_{D1}$	$\mathrm{P}^{+}_{D1}\mathrm{Phe}^{-}_{D1}$	β
0	14720	14650	14460	14560	14490	14480	14372	13972	2681

Supplementary Table S1: State energies in wavenumbers for PSIIRC photocell, reorganisation energy is not included.

	P_{D1}	P_{D2}	Chl_{D1}	Chl_{D2}	Phe_{D1}	Phe_{D2}	$\mathrm{Chl}^{+}_{D1}\mathrm{Phe}^{-}_{D1}$	$\mathrm{P}^{+}_{D1}\mathrm{Phe}^{-}_{D1}$
P_{D1}	-	150	-42	-55	-6	17	0	0
P_{D2}		-	-56	-36	20	-2	0	0
Chl_{D1}			-	7	46	-4	70	0
Chl_{D2}				-	-5	37	0	0
Phe_{D1}					-	-3	70	0
Phe_{D2}						-	0	0
$\mathrm{Chl}^{+}_{D1}\mathrm{Phe}^{-}_{D1}$							-	40
$\mathrm{P}^{+}_{D1}\mathrm{Phe}^{-}_{D1}$								-

Supplementary Table S2: Electronic couplings between PSIIRC core chromphores and CT states in wavenumbers.

Spectral Densities

The parameters for the low energy phonon environment, described using a Drude spectral density $J_D(\omega)$ (see Eq. 1 in the main paper) are $\lambda_D = 35\mathrm{cm}^{-1}$ and $\Omega_D = 40\mathrm{cm}^{-1}$, which are the reorganisation energy and cut-off frequency respectively[21,26]. The parameters for the high energy modes used in the full PSIIRC spectral density $J(\omega)$ are given in Supplementary Table S3[21,28] for which a damping constant $\gamma_j = 10\mathrm{cm}^{-1}$ is used for all modes[28] and the reorganisation energy associated with each mode is given by $\lambda_j = \omega_j s_j$. The frequencies, Huang-Rhys factors and damping constants for the broadened modes in spectral densities $J_1(\omega)$ and $J_2(\omega)$, used to approximate the 48 underdamped modes in non-perturbative calculations, are $\omega_1 = 342\mathrm{cm}^{-1}$, $s_1 = 0.4$, $\gamma_1 = 100\mathrm{cm}^{-1}$, and $\omega_2 = 742\mathrm{cm}^{-1}$, $s_2 = 0.32$, $\gamma_2 = 100\mathrm{cm}^{-1}$. The reorganisation energies for the broadened modes are given by $\lambda_i = \omega_i s_i$. To quantify the stronger coupling of the CT states to the bath, the reorganisation energies and line broadening functions of the primary (I) and secondary (α) CT states were scaled using values of $\nu_I = 3$ and $\nu_\alpha = 4$ respectively[26].

Supplementary Note 2: Theoretical Methods for Electronic Dynamics

The different parameter regimes found within PSIIRC systems make it a challenging computational task to accurately describe the exciton and charge transfer dynamics in a self-consistent manner. Two frameworks for the photocell

ω_j	s_j	ω_j	s_j	ω_j	s_j	ω_j	s_j
97	0.0371	604	0.0034	1143	0.0303	1354	0.0057
138	0.0455	700	0.005	1181	0.0179	1382	0.0067
213	0.0606	722	0.0074	1190	0.0084	1439	0.0067
260	0.0539	742	0.0269	1208	0.0121	1487	0.0074
298	0.0488	752	0.0219	1216	0.0111	1524	0.0067
342	0.0438	795	0.0077	1235	0.0034	1537	0.0222
388	0.0202	916	0.0286	1252	0.0051	1553	0.0091
425	0.0168	986	0.0162	1260	0.0064	1573	0.0044
518	0.0303	995	0.0293	1286	0.0047	1580	0.0044
546	0.0030	1052	0.0131	1304	0.0057	1612	0.0044
573	0.0094	1069	0.0064	1322	0.0202	1645	0.0034
585	0.0034	1110	0.0192	1338	0.0037	1673	0.001

Supplementary Table S3: Frequencies (cm^{-1}) and Huang-Rhys factors for 48 modes of PSIIRC

model are considered here: (i) A hybrid model where the exciton dynamics is described using the non-perturbative hierarchical equations of motion (HEOM). (ii) A Pauli master equation where the exciton dynamics is described using modified Redfield theory. Both these frameworks share a common description of the incoherent rates for charge transfer, optical excitation and coupling to leads which can be obtained from a Lindblad dissipator. More detail is given on the theoretical basis of these frameworks below.

Hamiltonian Dynamics

The Hamiltonian of the system and bath is $H = H_{el} + H_B + H_I$. The electronic Hamiltonian $H_{el} = \sum_i |i\rangle\langle i| + \sum_{ij} T_{ij}(|i\rangle\langle j| + |j\rangle\langle i|)$ is given in Supplementary Tables S1 and S2. The exciton states and associated energies are given in Supplementary Table S4. Additionally, $H_B = \sum_k \hbar\omega_k b_k^\dagger b_k$ is the bath Hamiltonian describing a continuum of bosonic modes and $H_I = \sum_{ki} g_{ik}|i\rangle\langle i|(b_k^\dagger + b_k)$ defines the interaction between the system and bath. The bath is characterised by a spectral density $J(\omega) = \sum_k g_k^2 \delta(\omega - \omega_k)$ for which several forms are investigated in the main paper (see Eqs. 1 and 2). The bath is assumed identical for each site apart from a scaling for the CT states taking into account their stronger coupling to the bath. Different open quantum systems approaches, depending on the framework considered, are used to determine the dynamics of the electronic system under the influence of the bath.

	P_{D1}	P_{D2}	Chl_{D1}	Chl_{D2}	Phe_{D1}	Phe_{D2}	ϵ (cm^{-1})
X_1	-0.029	0.251	**0.8**	-0.018	-0.541	0.048	14412.5
X_2	-0.185	0.057	-0.061	-0.404	0.04	**0.891**	14459.7
X_3	0.265	-0.145	0.56	0.112	**0.754**	0.12	14517.0
X_4	**-0.558**	**0.735**	-0.007	0.116	0.345	-0.126	14540.8
X_5	0.134	0.096	-0.121	**0.876**	-0.134	0.416	14565.0
X_6	**-0.752**	**-0.603**	0.165	0.209	-0.003	-0.012	14864.0

Supplementary Table S4: Site amplitudes and associated energies for the six exciton states of the core PSIIRC chromophores. Bold amplitudes indicate the chromophore on which the exciton is quasi-localized. ϵ is the state energy not including reorganisation energy.

Charge Transfer: Förster Theory

Primary and secondary charge separation were modeled using Förster theory[42,43] (equivalent to Marcus rates in the excited state) which is perturbative with respect to the electronic coupling between states and describes incoherent electron transfer events. The Förster rate expression has the general form

$$k_{ab} = |V_{ab}|^2 S_{ab}, \tag{S1}$$

where V_{ab} is the electronic coupling between states a and b and S_{ab} is the spectral overlap between these states. This overlap can be expressed equivalently in both the time and frequency domain

$$S_{ab}^{(time)} = 2\mathcal{R}e \int_0^\infty dt e^{i\omega_{ab}t} e^{-i(\lambda_a+\lambda_b)t} e^{-(g_a(t)+g_b(t))} \tag{S2}$$

$$S_{ab}^{(freq)} = \frac{1}{2\pi}\int_{-\infty}^{\infty} d\omega \bar{D}_a(\omega) D_b(\omega), \tag{S3}$$

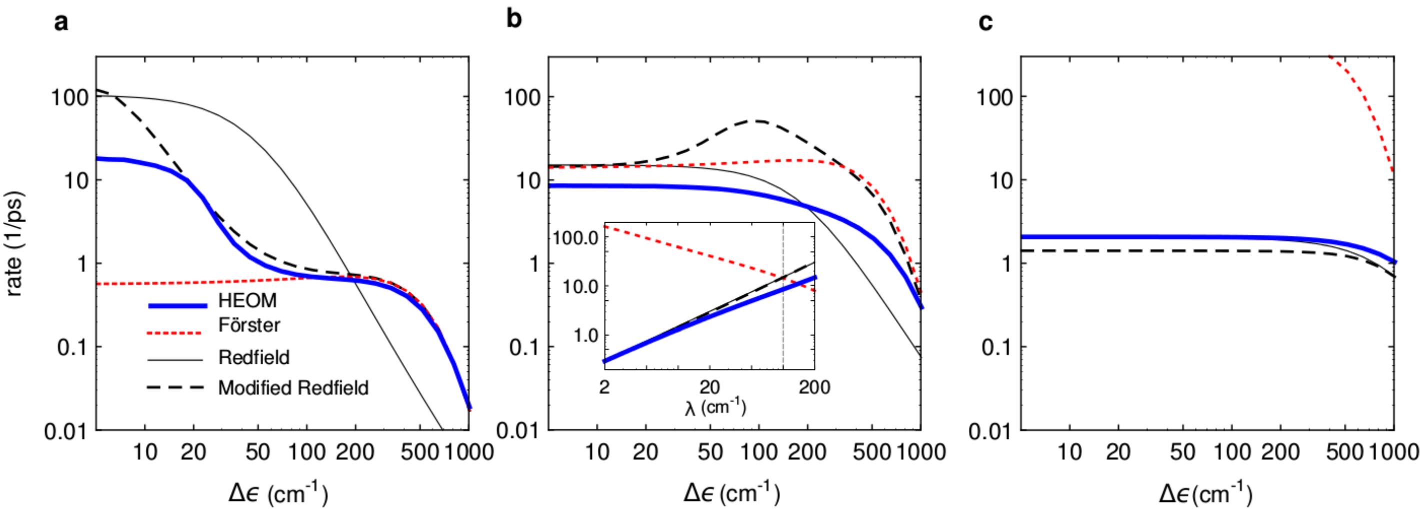

Supplementary Figure S1: Comparison of perturbative theories with the hierarchical equations of motion for downhill transfer rates in a dimer system[70]. The electronic system Hamiltonian is $H = (\Delta\epsilon/2)\sigma_z + V\sigma_x$ which is interacting with a Drude spectral density with reorganisation energy $\lambda = 100\text{cm}^{-1}$ and cutoff frequency $\Omega_c = 53\text{cm}^{-1}$. The rates are calculated for different electronic coupling strengths (a) V $= 20\text{cm}^{-1}$, (b) V $= 100\text{cm}^{-1}$ and (c) V $= 500\text{cm}^{-1}$. The inset of (b) shows the dependence of transfer rate on reorganisation energy λ for an energy gap $\Delta\epsilon = 10\text{cm}^{-1}$.

where $D_a(\omega) = 2\mathcal{R}e\int_0^\infty dt e^{i\omega t}e^{-i\omega_a t - i\lambda_a t - g_a(t)}$ and $\bar{D}_a(\omega) = 2\mathcal{R}e\int_0^\infty dt e^{i\omega t}e^{-i\omega_a t + i\lambda_a t - g_a^*(t)}$ are the absorption and fluorescence lineshapes respectively. The line broadening functions are defined $g(t) = g_D(t) + \sum_j g_j(t)$ where $g_D(t)$ and $g_j(t)$ are the line broadening functions for the Drude mode and the jth underdamped mode respectively. For a Drude spectral density $g_D(t) = \frac{c_0^D}{\Omega_D^2}(e^{-\Omega_D t} + \Omega_D t - 1) + \sum_{k=1}^\infty \frac{c_k^D}{\nu_k^2}(e^{-\nu_k t} + \nu_k t - 1)$ where $c_0^D = \lambda_D\Omega_D(\cot(\frac{\beta\Omega_D}{2}) - i)$ and $c_k^D = \frac{4\lambda_D\Omega_D}{\beta}(\frac{\nu_k}{\nu_k^2 - \Omega_D^2})$. For an underdampded mode $g_j(t) = \sum_{+,-}\frac{c_{0j}^{\pm}}{\nu_{j\pm}^2}(e^{-\nu_{j\pm}t} + \nu_{j\pm}t - 1) + \sum_{k=1}^\infty \frac{c_{kj}}{\nu_k^2}(e^{-\nu_k t} + \nu_k t - 1)$, where $c_{0j}^{\pm} = \pm i\frac{\lambda_j\omega_j^2}{2\zeta_j}(\cot(\frac{\beta\nu_\pm}{2}) - i)$, $c_{kj} = \frac{-4\lambda_j\gamma_j\omega^2}{\beta}(\frac{\nu_k}{(\omega_j^2+\nu_k^2)^2 - (\gamma_j^2\nu_k^2)})$, $\nu_{j\pm} = \frac{\gamma_j}{2} \pm i\zeta_j$ and $\zeta_j = \sqrt{\omega_j^2 - \frac{\gamma_j^2}{4}}$. The summations over Matsubara frequencies $\nu_k = \frac{2\pi}{\beta}k$ can usually can truncated at a suitable k depending on the temperature.

For primary charge transfer we have transfer from an exciton state (X) to the primary CT state (I). In this case the electronic coupling between X and I is $V_{XI} = \sum_{n\in X} c_n(X)V_{nI}$ where $c_n(X)$ is the amplitude of site n in exciton X and V_{nI} is the electronic coupling between site n and CT state I. The exciton reorganisation energy has the form $\lambda_X = \sum_i |c_i(X)|^4\lambda$ and similarly the line broadening function $g_X(t) = \sum_i |c_i(X)|^4 g(t)$ where $c_i(X)$ are the amplitudes of exciton X at site i and λ and $g(t)$ are the site reorganisation energy and line broadening function. The primary CT reorganisation energy and line broadening function are $\lambda_I = \nu_I\lambda$ and $g_I(t) = \nu_I g(t)$.

Secondary charge transfer rates are calculated in a similar way though the reorganisation energies and line broadening functions of both the donor and acceptor are now scaled.

Justification of Perturbative Treatment for Charge Transfer Dynamics

The parameter regime for primary charge separation in the Chl_{D1} pathway is such that a perturbative approach to the dynamics using Förster theory is a good approximation to the exact dynamics. In the site basis the electronic coupling between Chl_{D1} and Phe_{D1} and the primary CT state is 70cm^{-1} while the CT reorganisation energy ranges from 105cm^{-1} for a Drude spectral density $J_D(\omega)$ to 1769cm^{-1} for the full spectral density $J(\omega)$. A comparison of transfer rates calculated using HEOM and Förster theory is given in Supplementary Fig. S1[70], which shows downhill transfer rates for a dimer system in various parameter regimes. The relevant regime comparable to primary CT in PSIIRC is the right-hand side of plot **a**, where the electronic coupling is less than both the reorganisation energy and the energy gap. In this region, Förster theory and HEOM give transfer rates in close agreement. The small electronic coupling and large energy gap mean transfer must occur through transient resonant states of the system rather than through coherent evolution.

Incoherent Excitation and Coupling to Leads

For the incoherent rates of electron hopping between the source lead of the system a value of $\Gamma_L = 201.6\text{cm}^{-1}$ was used. The rate to hop from the system into the drain lead (Γ_R) was varied for the current and Fano factor calculations as a function of voltage.

For calculations varying voltage with a constant excitation rate, the value $\gamma_{ex} = \gamma n = 75\text{cm}^{-1}$ where $\gamma = 0.00125\text{cm}^{-1}$ and $n = 60000$ were used to simulate excitation by concentrated solar radiation[37]. Satisfying detailed balance the deexcitation rate $\gamma_{deex} = \gamma(n+1)$.

Hybrid Model: Hierarchical Equations of Motion

The exciton relaxation dynamics in photosynthetic systems can be described using the non-perturbative hierarchical equations of motion (HEOM)[34,35,36]. This treats the exciton-vibrational dynamics exactly but is computationally expensive and requires the hierarchy to be truncated at some suitable level. In order to include additional incoherent processes in the photocell dynamics a hybrid model must be used. In this case a Lindblad dissipator is included to capture the coupling of the excitons to the rest of the photocell[68]. In particular the dissipator describes the excitation of the system from the ground to exciton manifold, primary and secondary charge transfer, and coupling to the leads.

Due to the numerical difficulty of describing the system-bath dynamics the full PSIIRC spectral density cannot be included in this framework. Instead, a Drude mode and a broadened underdamped mode (see Fig. 2 from the main paper) overlapping the exciton energy gaps is used to approximate the full spectral density. In the main paper the photocell current and power output for this approximate spectral density are compared with a photocell having just a Drude bath.

The infinite hierarchy of coupled differential equations including the Lindblad dissipator has the form:

$$\begin{aligned}\dot{\sigma}^{(\boldsymbol{n},\boldsymbol{m}^-,\boldsymbol{m}^+)} = \; & [\mathcal{L} - \boldsymbol{n}\boldsymbol{\nu}_D - \frac{1}{2}(\boldsymbol{m}^+ + \boldsymbol{m}^-)\boldsymbol{\nu}_0 + i(\boldsymbol{m}^- - \boldsymbol{m}^+)\boldsymbol{\zeta}_0 - \Xi_{TC}]\sigma^{(\boldsymbol{n},\boldsymbol{m}^-,\boldsymbol{m}^+)} \\ & + i\sum_i^N \sqrt{(n_i+1)c_i^D} V_i^\times \sigma^{(\boldsymbol{n}_{i+1},\boldsymbol{m}^-,\boldsymbol{m}^+)} \\ & + i\sum_i^N \sqrt{(m_i^-+1)c_i^-} V_i^\times \sigma^{(\boldsymbol{n},\boldsymbol{m}^-_{i+1},\boldsymbol{m}^+)} \\ & + i\sum_i^N \sqrt{(m_i^++1)c_i^+} V_i^\times \sigma^{(\boldsymbol{n},\boldsymbol{m}^-,\boldsymbol{m}^+_{i+1})} \\ & + \sum_i^N \sqrt{\frac{n_i}{|c_i^D|}}\Theta_i \sigma^{(\boldsymbol{n}_{i-1},\boldsymbol{m}^-,\boldsymbol{m}^+)} + \sum_i^N \sqrt{\frac{m_i^-}{|c_i^-|}}\Theta_i^- \sigma^{(\boldsymbol{n},\boldsymbol{m}^-_{i-1},\boldsymbol{m}^+)} + \sum_i^N \sqrt{\frac{m_i^+}{|c_i^+|}}\Theta_i^+ \sigma^{(\boldsymbol{n},\boldsymbol{m}^-,\boldsymbol{m}^+_{i-1})} \end{aligned} \tag{S4}$$

where $\sigma^{(\boldsymbol{n},\boldsymbol{m}^-,\boldsymbol{m}^+)}$ are auxiliary density matrices indexed by the vectors $\boldsymbol{n} = \{n_1, n_2, ..., n_N\}$ to account for the Drude part of the spectral density and $\boldsymbol{m}^- = \{m_1^-, m_2^-, ..., m_N^-\}, \boldsymbol{m}^+ = \{m_1^+, m_2^+, ..., m_N^+\}$ for the underdamped mode. The system density matrix $\rho_s(t)$ is given by the matrix indexed by zero vectors, $\sigma^{(\boldsymbol{0},\boldsymbol{0},\boldsymbol{0})}$. $\boldsymbol{n}_{i\pm1}$ indicates the vector $\boldsymbol{n}$ with element i $\rightarrow n_i \pm 1$ with similar definitions for $\boldsymbol{m}^{\pm}_{i\pm1}$.

The coherent system dynamics and incoherent rates for charge transfer, excitation and coupling to the leads are accounted for within the Liouvillian $\mathcal{L}\bullet = -\frac{i}{\hbar}[H_{el}, \bullet] + \mathcal{D}(\bullet)$ where H_el is the electronic Hamiltonian and $\mathcal{D}(\sigma) = \sum_p \gamma_p[a_p\sigma a_p^\dagger - \frac{1}{2}\{a_p^\dagger a_p \sigma\}]$ is a Lindblad dissipator. γ_p are the incoherent rate for coupling between the exciton states and the rest of the photocell with $a_p^\dagger(a_p)$ the associated jump operators.

The vectors $\boldsymbol{\nu}_D, \boldsymbol{\nu}_0$ and $\boldsymbol{\zeta}_0$ contain the Drude cutoff frequency Ω_D, the mode damping constant γ_0 and the quantity $\zeta_0 = \sqrt{\omega_0^2 - \frac{1}{4}\gamma_0^2}$ respectively, for each site of the system.

The hierarchy above is given in the rescaled form which allows for efficient numerical computation of the time dynamics and steady state. The coefficients c_i^D, c_i^- and c_i^+ are the leading coefficients of the exponential expansion of the bath correlation functions for Drude (D) and underdamped Brownian oscillator ($\pm$) spectral densities. Matsubara terms were not explicitly considered as they had little effect on the dynamics at 300K and increased the cost of computation. However a temperature correction term which approximates the effect of the Matsubara frequencies was included: $\Xi_{TC} = \sum_i \sum_{k=1}^\infty \Psi_{ik} V_i^\times V_i^\times$ where $\sum_{k=1}^\infty \Psi_{ik} = \frac{2\lambda_D - \beta\Omega_D\lambda_D\cot(\frac{\beta\Omega_D}{2})}{\beta\Omega_D} + \frac{\lambda_0}{2\zeta_0}(\frac{\sin(\frac{\beta\gamma_0}{2}) + \gamma_0\sinh(\beta\zeta_0)}{\cos(\frac{\beta\gamma_0}{2}) - \cosh(\beta\zeta_0)}) + \frac{2\gamma_0}{\beta\omega_0^2}$.

The operators for coupling to the lower tiers are

$$\Theta_i = i\lambda_D\Omega_D \arctan(\frac{\beta\Omega_D}{2})V_i^\times + \lambda_D\Omega_D V_i^\circ \tag{S5}$$

$$\Theta_i^\pm = \mp i\frac{\lambda_0\,\omega_0^2}{2\zeta_0}\arctan\{\frac{i\beta}{4}(\gamma_0 \pm 2i\zeta_0)\}V_i^\times \pm i\frac{\lambda_0\,\omega_0^2}{2\zeta_0}V_i^\circ \tag{S6}$$

with $V^\times\bullet = [V_i, \bullet]$ and $V^\circ\bullet = \{V_i, \bullet\}$ the superoperators representing commutator and anticommutator relations involving site i system-bath coupling operator $V_i = |i\rangle\langle i|$.

Pauli Master Equation: Modified Redfield Theory

An alternative framework used to model the PSIIRC photocell involves a Pauli master equation where modified Redfield theory is used to calculate the exciton relaxation dynamics[26]. The other incoherent rates for excitation, charge transfer and coupling to leads are the same as in the hybrid model framework. The reduced computational cost of the Pauli master equation allowed an investigation of the effect of the full PSIIRC spectral density on the behaviour of the photocell and also allowed the electron counting statistics of the photocell to be studied.

Modified Redfield theory[43,69] describes population-to-population transfer among strongly coupled groups of chromophores. The transfer rates take into account the strong coupling to the phonon-bath but treat as a perturbation the off-diagonal coupling in the exciton basis i.e. $H' = \sum_{k\neq k'} |k\rangle H^{el-ph}_{kk'} \langle k'|$, where $|k\rangle$ are exciton states. The electron-phonon coupling matrix elements are given by $H^{el-ph}_{kk'} = \sum_{n=1}^{N} a_{kk'}(n) u_n$ where $a_{kk'}(n)$ is the spatial overlap of excitons $|k\rangle$ and $|k'\rangle$ at site n. It is clear that modified Redfield should work well for systems in which we have quasi-localised excitons (ie. $a_{kk'}(n)$ is small), which is the situation for the Chl_{D1} pathway in PSIIRC. The Markovian transfer rate between excitons a and b is given by the expression

$$\begin{aligned} k_{ab} = 2\int_0^\infty & dt \exp(i\omega_{ab}t - i(\lambda_{aaaa} + \lambda_{bbbb} - 2\lambda_{bbaa})t - g_{aaaa}(t) - g_{bbbb}(t) + 2g_{bbaa}(t)) \\ & \times (\ddot{g}_{baba}(t) - (\dot{g}_{babb} - \dot{g}_{baaa} + 2i\lambda_{babb})^2) \end{aligned} \tag{S7}$$

where the reorganisation energies and line broadening functions are given by $\lambda_{pqrs} = \sum_i c_i(p)c_i(q)c_i(r)c_i(s)\lambda_i$ and $g_{pqrs}(t) = \sum_i c_i(p)c_i(q)c_i(r)c_i(s)g_i(t)$. $c_i(p)$ is the amplitude of exciton p on site i, λ_i is the reorganisation energy of site i and $g_i(t)$ is the line broadening function for site i.

The rates for coupling to the leads, excitation and charge transfer are treated equivalently in both frameworks we consider. The Pauli master equation can be obtained from a Lindblad dissipator having the form $\mathcal{D}(\rho) = \sum_p \gamma_p [a_p \rho a_p^\dagger - \frac{1}{2}\{a_p^\dagger a_p \rho\}]$. The jump operators $a_p (a_p^\dagger)$ causing population transfer between two states of the system give contributions to the time evolution of both the populations and coherences. Since the populations and coherences are decoupled from each other in the Lindblad formalism the coherences can be neglected and a master equation for the populations is obtained. This is given in the form of the Liouville space operator in Supplementary Eq. S8. The diagonal elements of the Pauli matrix have been omitted for compactness, but each column must sum to zero in order to satisfy a trace preserving time evolution. The rates k_{XY} where $X, Y = 1, .., 6$ are the modified Redfield rates for transfer between excitons. The additional rates have been discussed in the previous sections where $k_{XI(IX)}$ and $k_{I\alpha(\alpha I)}$ are Förster rates for primary and secondary charge separation and rates $\gamma n, \gamma(n+1), \Gamma_L$ and Γ_R are for optical excitation and coupling to leads.

$$\begin{pmatrix} \dot{\rho}_{gg} \\ \dot{\rho}_{X1} \\ \dot{\rho}_{X2} \\ \dot{\rho}_{X3} \\ \dot{\rho}_{X4} \\ \dot{\rho}_{X5} \\ \dot{\rho}_{X6} \\ \dot{\rho}_{II} \\ \dot{\rho}_{\alpha\alpha} \\ \dot{\rho}_{\beta\beta} \end{pmatrix} = \begin{pmatrix} - & \gamma(n+1) & 0 & 0 & 0 & 0 & 0 & 0 & 0 & \Gamma_L \\ \gamma n & - & k_{21} & k_{31} & k_{41} & k_{51} & k_{61} & k_{I1} & 0 & 0 \\ 0 & k_{12} & - & k_{32} & k_{42} & k_{52} & k_{62} & k_{I2} & 0 & 0 \\ 0 & k_{13} & k_{23} & - & k_{43} & k_{53} & k_{63} & k_{I3} & 0 & 0 \\ 0 & k_{14} & k_{24} & k_{34} & - & k_{54} & k_{64} & k_{I4} & 0 & 0 \\ 0 & k_{15} & k_{25} & k_{35} & k_{45} & - & k_{56} & k_{I5} & 0 & 0 \\ 0 & k_{16} & k_{26} & k_{36} & k_{46} & k_{56} & - & k_{I6} & 0 & 0 \\ 0 & k_{1I} & k_{2I} & k_{3I} & k_{4I} & k_{5I} & k_{6I} & - & k_{\alpha I} & 0 \\ 0 & 0 & 0 & 0 & 0 & 0 & 0 & k_{I\alpha} & - & 0 \\ 0 & 0 & 0 & 0 & 0 & 0 & 0 & 0 & \Gamma_R & - \end{pmatrix} \begin{pmatrix} \rho_{gg} \\ \rho_{X1} \\ \rho_{X2} \\ \rho_{X3} \\ \rho_{X4} \\ \rho_{X5} \\ \rho_{X6} \\ \rho_{II} \\ \rho_{\alpha\alpha} \\ \rho_{\beta\beta} \end{pmatrix} \tag{S8}$$

Supplementary Note 3: Comparison of Theoretical Frameworks for PSIIRC Photocell Dynamics and Steady States

The two frameworks we consider in the main paper are compared here to justify the use of modified Redfield theory to describe exciton relaxation in the Pauli master equation framework. In Supplementary Fig. S2 the transient dynamics of the full photocell for the different spectral densities is presented, with the exciton populations summed to simplify the plot. For the Drude ($J_D(\omega)$) and single underdamped Browian oscillator ($J_1(\omega)$) spectral densities the hybrid model dynamics is well approximated by the Pauli master equation at both the initial stages and at longer times where the systems are relaxing towards a steady state. The Pauli dynamics with the full spectral density ($J(\omega)$) is shown for completeness. The dynamics of exciton coherences as calculated with the hybrid model are shown in Supplementary Fig. S3. This shows the exciton coherences are small in amplitude and decay quickly towards a steady state close to zero. The use of modified Redfield which does not carry information about coherences between exciton states is justified on these grounds.

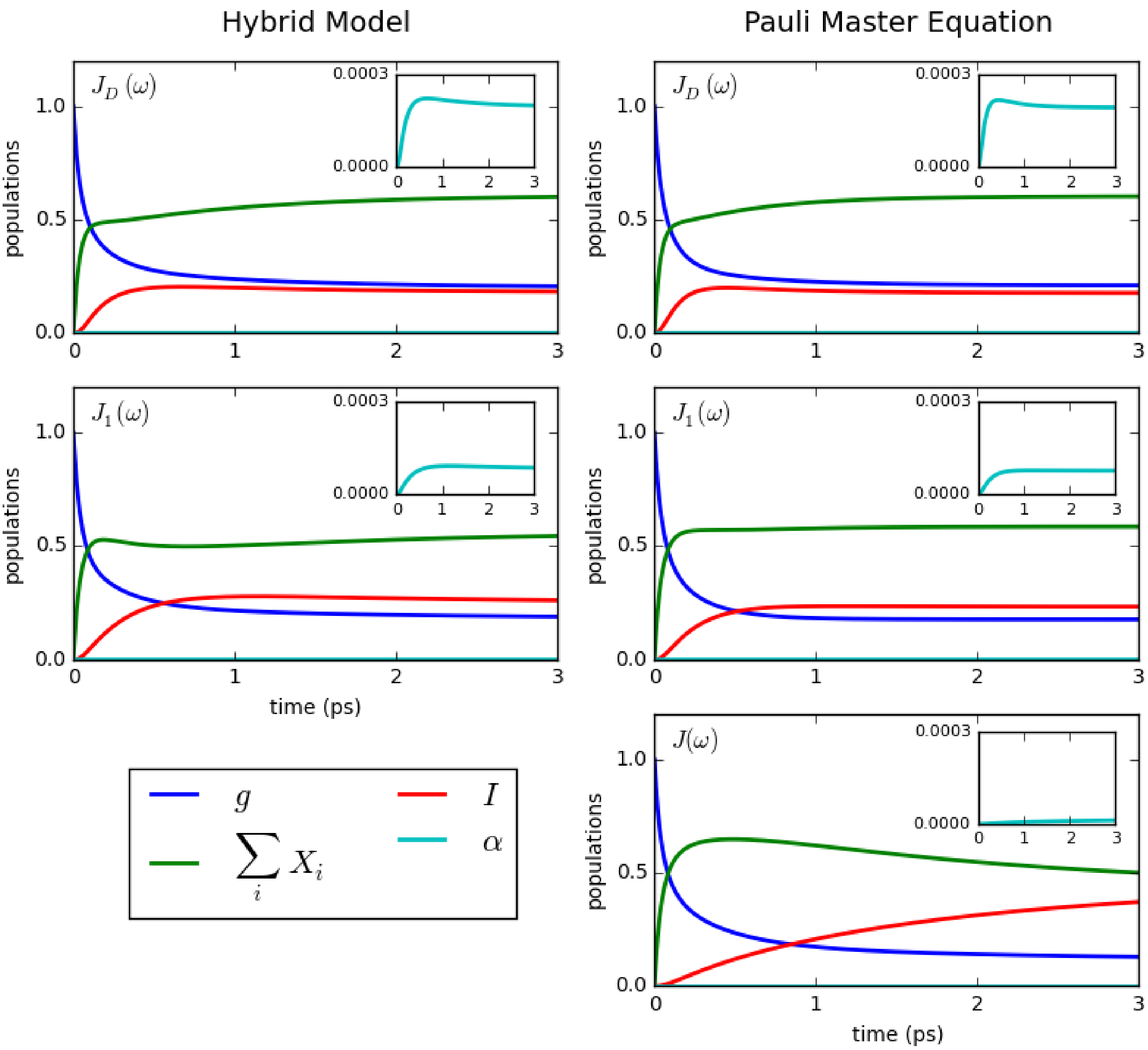

Supplementary Figure S2: Comparison of PSIIRC photocell population dynamics using the hybrid model and Pauli master equation. The exciton populations (X_i) have been summed to simplify the plots and the populations of the secondary CT state (α) are shown in the insets. The population of state β is not shown.

Additionally the steady states calculated by the two theories are compared in Supplementary Fig. S4 which shows the long time behaviour of the photocell. The steady state plays an important role in the counting statistics calculations. There is a close agreement between the two frameworks which suggests that calculations using the Pauli master equation should give results representative of the hybrid model behaviour.

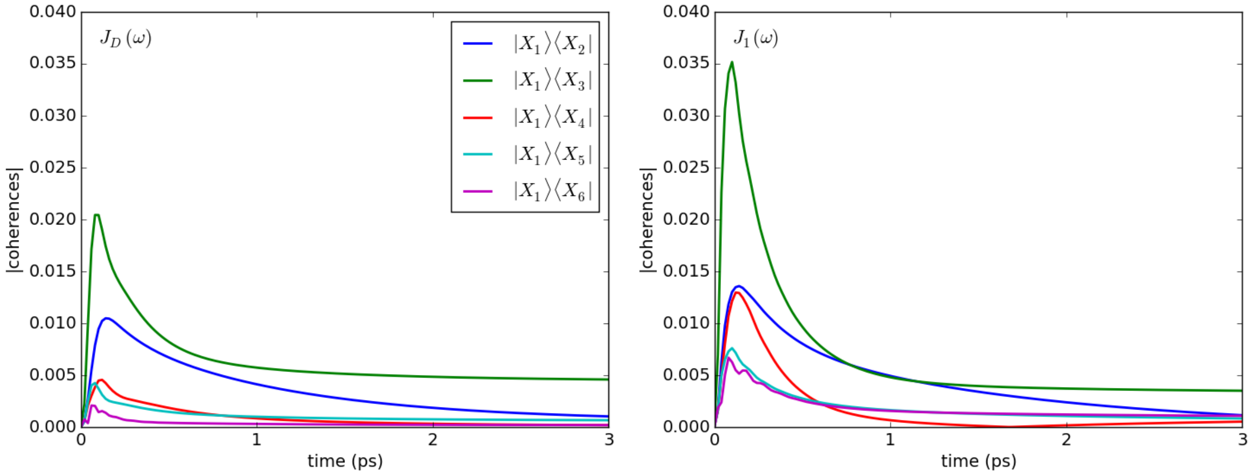

Supplementary Figure S3: Exciton coherence dynamics within the hybrid model for spectral densities $J_D(\omega)$ and $J_1(\omega)$. Only coherences between exciton X_1 and the other excitons are shown.

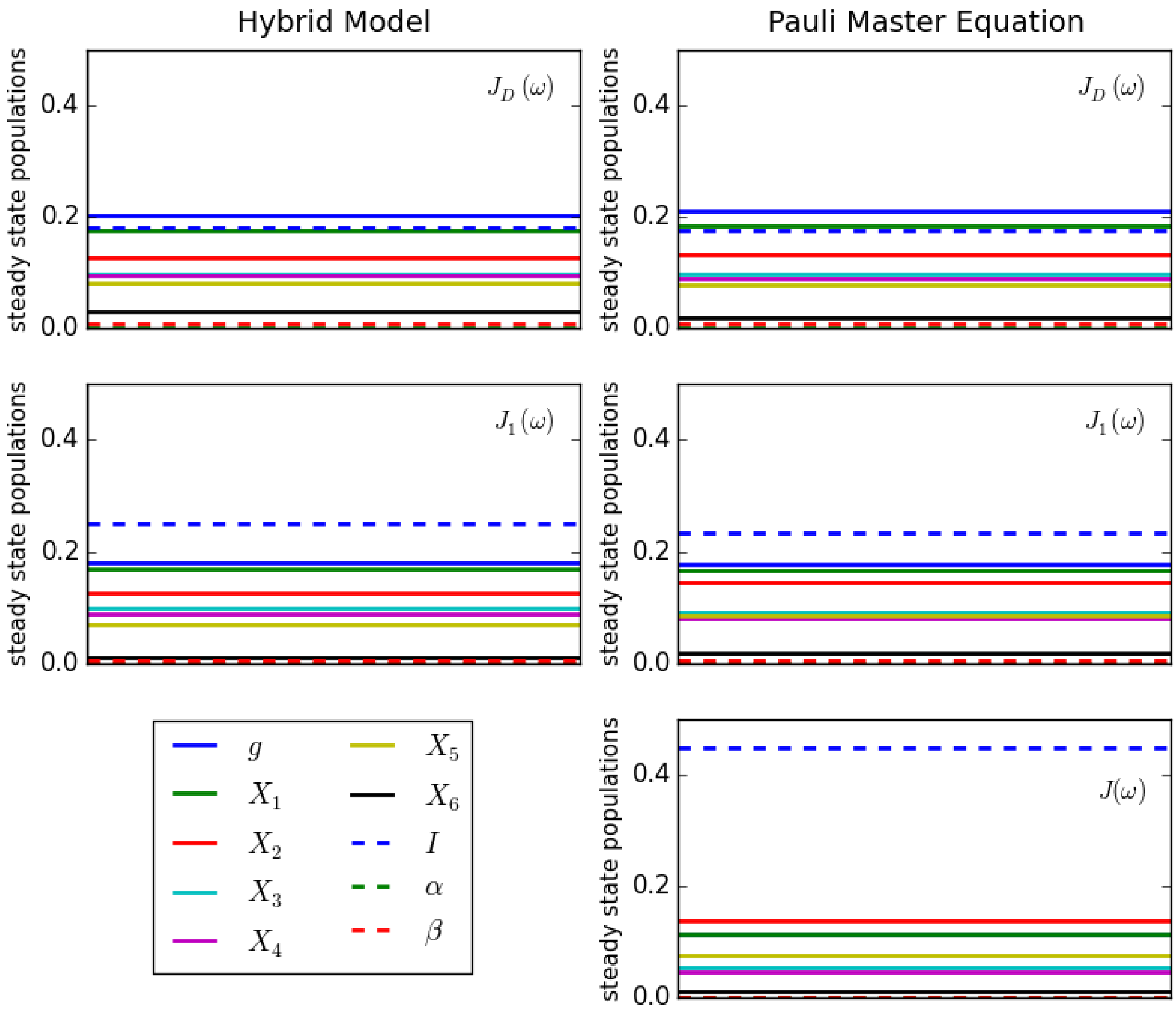

Supplementary Figure S4: Comparison of PSIIRC photocell steady states using the hybrid model and Pauli master equation for different spectral densities.

Supplementary Note 4: Reduction of Förster Rates on Increasing Reorganisation Energy

Since charge transfer occurs between excitons and a primary CT state and then between the primary and secondary CT states there is an asymmetry of reorganisation energies between each donor-acceptor pair due to the scaling of the bath coupling for the CT states. Supplementary Fig. S5 shows donor fluorescence and acceptor absorption lineshapes for forward primary and secondary charge transfer. For primary CT, the fluorescence lineshape $\bar{D}_{X1}(\omega)$ for the exciton quasi-localised at site Chl_{D1} and the absorption lineshape $D_I(\omega)$ for the primary CT state are shown. Similarly for secondary CT, the fluorescence lineshape $\bar{D}_I(\omega)$ for the primary CT state and the absorption lineshape $D_\alpha(\omega)$ for the secondary CT state are shown. The position of the absorption and fluorescence lineshapes depends on the reorganisation energy of the states involved. As modes are added to the spectral density the reorganisation energy of the states increase but the reorganisation energy of the acceptor state (for forward transfer) increases much more rapidly. Despite broadening of the lineshapes, the absorption peaks move away from the fluorescence peaks reducing the overlap between them and therefore decreasing the Förster rate between the exciton and the CT state. The lineshapes shown below are not individually normalized in order to show the relative amplitudes at which the lineshapes enter the equation for the overlap $S_{ab}^{(freq)}$.

	$J_D(\omega)$	$J_1(\omega)$	$J(\omega)$
$X_1 \to I$	0.01122	0.00360	0.00139
$X_2 \to I$	0.01123	0.00353	0.00124
$X_3 \to I$	0.01114	0.00432	0.00149
$X_4 \to I$	0.01071	0.00447	0.00151
$X_5 \to I$	0.01023	0.00415	0.00135
$X_6 \to I$	0.00215	0.00475	0.00149
$I \to \alpha$	0.00676	0.00206	0.00021

	$J_D(\omega)$	$J_1(\omega)$	$J(\omega)$
X_1	17.51	85.95	295.019
X_2	23.048	113.132	388.32
X_3	14.939	73.332	251.707
X_4	14.116	69.288	237.829
X_5	21.67	106.367	365.099
X_6	15.91	78.093	268.051
I	105	515.4	1769.087
α	140	687.2	2358.782

Supplementary Table S5: (Left) Spectral overlaps for the forward charge transfer between exciton states and the primary CT state, and between the primary and secondary CT states for different spectral densities at 300K. (Right) Reorganisation energies in wavenumbers (cm^{-1}) for excitons and CT states for different spectral densities at 300K.

Supplementary Note 5: Theory of Full Counting Statistics

Here we outline a derivation of the zero-frequency noise for both the hybrid and Pauli models used in the main paper. The derivation starts from a general time-local master equation $\dot{\sigma}(t) = \mathcal{M}\sigma(t)$ where the state of the system $\sigma(t)$ is propagated through time by an operator $\mathcal{M}$. For the hybrid model the state of the system is a vector of auxiliary density matrices with the hierarchy matrix acting as a time propagator. For the Pauli model the state of the system is a vector of state populations with the associated rate matrix given in Eq. S8. The counting statistics are generated by singling out the counting transition $|\alpha\rangle \to |\beta\rangle$ with a counting field χ to obtain a perturbed time propagator $\mathcal{M}(\chi) = \mathcal{M} + (e^{i\chi} - 1)\mathcal{M}_J$. $\mathcal{M}_J$ is a jump matrix describing transitions between the systems and the drain lead. In the hybrid model $\mathcal{M}_J$ is the part of the Lindblad dissipator describing the counting transition in the top level of the hierarchy only. A recursive scheme[44] can then be followed which generates zero-frequency current cumulants up to any order and expresses them in terms of the jump matrix $\mathcal{M}_J$ and a pseudo-inverse $\mathcal{R}$ of the time propagator. The current cumulants are obtained from the maximal eigenvalue $\lambda_0(\chi)$ of $\mathcal{M}(\chi)$ which becomes $\lambda_0(0) = 0$ and solves the eigenvalue problem

$$\mathcal{M}(\chi)|0(\chi)\rangle\rangle = \lambda_0(\chi)|0(\chi)\rangle\rangle, \tag{S9}$$

$|0(\chi)\rangle\rangle$ being the steady state left-eigenvector of $\mathcal{M}(\chi)$. The eigenvalue can be written

$$\lambda_0(\chi) = \langle\langle\tilde{0}|\Delta\mathcal{M}(\chi)|0(\chi)\rangle\rangle, \tag{S10}$$

where $\langle\langle\tilde{0}|\mathcal{L} = 0$ (steady state right-eigenvector acting on unperturbed generator), $\langle\langle\tilde{0}|0(\chi)\rangle\rangle = 1$ (normalization) and $\Delta\mathcal{M}(\chi) = (e^{i\chi} - 1)\mathcal{M}_J$ (perturbation).

Using the projectors $\mathcal{P} = \mathcal{P}^2 = |0\rangle\rangle\langle\langle\tilde{0}|$ (projector onto the steady state subspace of the system) and $\mathcal{Q} = \mathcal{Q}^2 = I - \mathcal{P}$ (projector onto the space orthogonal to the steady state) the steady state can be written

$$|0(\chi)\rangle\rangle = |0\rangle\rangle + \mathcal{Q}|0(\chi)\rangle\rangle. \tag{S11}$$

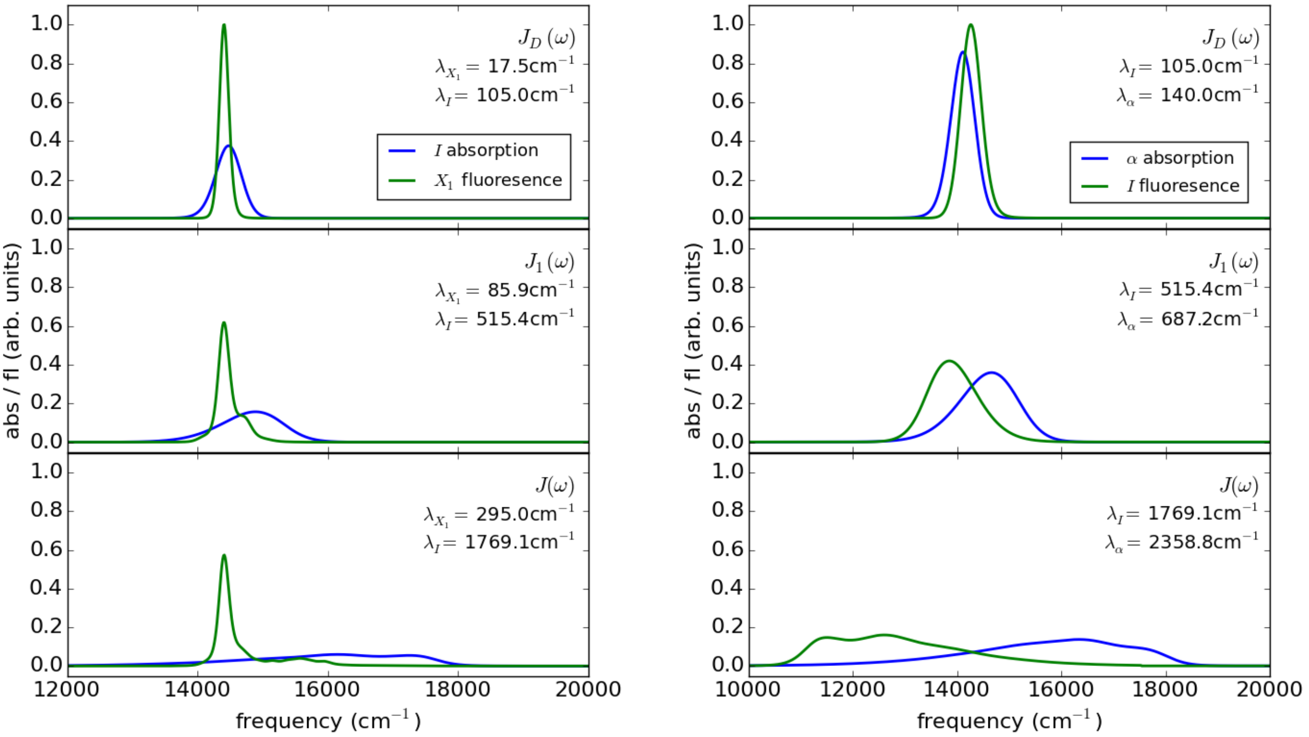

Supplementary Figure S5: Spectral lineshapes for different spectral densities at 300K. (Left) Exciton state (X_1) fluorescence and primary CT state (I) absorption lineshapes. (Right) Primary CT state (I) fluorescence and secondary CT state (α) absorption lineshapes.

Additionally using $\mathcal{M} = \mathcal{QMQ}$ the eigenvalue equation can be written

$$\mathcal{Q}\mathcal{M}(\chi)\mathcal{Q}|0(\chi)\rangle\rangle = [\lambda_0(\chi) - \Delta\mathcal{M}(\chi)]|0(\chi)\rangle\rangle. \tag{S12}$$

Introducing the pseudo-inverse $\mathcal{R} = \mathcal{Q}\mathcal{M}^{-1}\mathcal{Q}$ which is well defined since it is performed in the subspace orthogonal to the steady state. We then have

$$\mathcal{Q}|0(\chi)\rangle\rangle = \mathcal{R}[\lambda_0(\chi) - \Delta\mathcal{M}(\chi)]|0(\chi)\rangle\rangle, \tag{S13}$$

which on substituting in Eq. S11 we get

$$|0(\chi)\rangle\rangle = |0\rangle\rangle + \mathcal{R}[\lambda_0(\chi) - \Delta\mathcal{M}(\chi)]|0(\chi)\rangle\rangle. \tag{S14}$$

Equations S10 and S14 are the starting points for the recursive scheme. Expanding the zero eigenvalue $\lambda_0(\chi)$, steady state $|0(\chi)\rangle\rangle$ and the perturbation $\Delta\mathcal{M}(\chi)$ about $\chi = 0$ gives

$$\lambda_0(\chi) = \sum_{n=1}^{\infty} \frac{(i\chi)^n}{n!} \langle\langle I^n \rangle\rangle \tag{S15}$$

$$|0(\chi)\rangle\rangle = \sum_{n=0}^{\infty} \frac{(i\chi)^n}{n!} |0^{(n)}\rangle\rangle \tag{S16}$$

$$\Delta\mathcal{M}(\chi) = \sum_{n=1}^{\infty} \frac{(i\chi)^n}{n!} \mathcal{M}^{(n)} \tag{S17}$$

From this it is clear that the zero eigenvalue is equivalent to the cumulant generating function of the statistics. Substituting these expansions into Eqs. S10 and S14 give us the following expressions to calculate the cumulant to any order ($n = 1, 2, 3, ...$)

$$\langle\langle I^n \rangle\rangle = \sum_{m=1}^{n} \binom{n}{m} \langle\langle \tilde{0}|\mathcal{M}^{(n)}|0^{(n-m)}\rangle\rangle \tag{S18}$$

$$|0^{(n)}\rangle\rangle = \mathcal{R} \sum_{m=1}^{n} \binom{n}{m} [\langle\langle I^m \rangle\rangle - \mathcal{M}^{(m)}]|0^{(n-m)}\rangle\rangle \tag{S19}$$

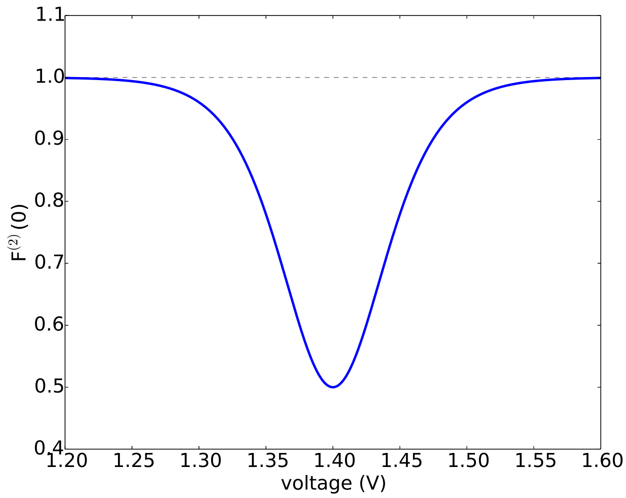

Supplementary Figure S6: Zero-frequency Fano factor versus voltage for a single resonant level. $E_0 = 11291.7cm^{-1}$ and $T = 300K$.

The first and second order cumulant which are used to obtain the numerical results later on are explicitly shown below

$$\langle\langle I^1 \rangle\rangle = \langle\langle \tilde{0}|\mathcal{M}^{(1)}|0\rangle\rangle \quad \text{(S20)}$$

$$\langle\langle I^2 \rangle\rangle = \langle\langle \tilde{0}|\mathcal{M}^{(2)} - 2\mathcal{M}^{(1)}\mathcal{R}\mathcal{M}^{(1)}|0\rangle\rangle \quad \text{(S21)}$$

It is useful to note that $\mathcal{M}^{(n)} = \mathcal{M}_J$ in the above since $\frac{\partial^n}{\partial(i\chi)^n}\Delta\mathcal{M}(\chi)|_{\chi=0} = \frac{\partial^n}{\partial(i\chi)^n}\mathcal{M}_J(e^{i\chi} - 1)|_{\chi=0}$.

Supplementary Note 6: Counting Statistics of a Single Resonant Level

Equation 6 from the main text is plotted in Supplementary Fig. S6 to show the voltage dependence of the zero-frequency Fano factor for electrons passing through a single resonant level. The derivation of Eq. 6 is outlined in the following.

The Liouvillian for the single resonant level system has the form[29,63]

$$\mathcal{L} = \begin{pmatrix} -\Gamma_L & \Gamma_R \\ \Gamma_L & -\Gamma_R \end{pmatrix}, \quad \text{(S22)}$$

from which the steady state populations of the empty and occupied states can be calculated as $\rho_{empty} = \frac{\Gamma_R}{\Gamma_L+\Gamma_R}$ and $\rho_{occupied} = \frac{\Gamma_L}{\Gamma_L+\Gamma_R}$. Substituting the expressions for the steady state populations into $eV = E_0 + k_B T \ln(\frac{\rho_{occupied}}{\rho_{empty}})$ leads to

$$\Gamma_R = \Gamma_L \exp(-(eV - E_0)/k_B T). \quad \text{(S23)}$$

This can be used with the expression for the Fano factor in terms of the rates Γ_L and Γ_R [29],

$$F^{(2)} = \frac{\Gamma_L^2 + \Gamma_R^2}{(\Gamma_L + \Gamma_R)^2}, \quad \text{(S24)}$$

to give the Fano factor as a function of V,

$$F^{(2)}(V) = \frac{1 + \exp[-2(eV - E_0)/k_B T]}{(1 + \exp[-(eV - E_0)/k_B T])^2}. \quad \text{(S25)}$$
